# Evaluation of TNF-α, IL-10 and IL-6 Cytokine Production and Their Correlation with Genotype Variants amongst Tuberculosis Patients and Their Household Contacts

**DOI:** 10.1371/journal.pone.0137727

**Published:** 2015-09-11

**Authors:** Lavanya Joshi, Meenakshi Ponnana, Ramya Sivangala, Lakshmi Kiran Chelluri, Prathiba Nallari, Sitaramaraju Penmetsa, Vijayalakshmi Valluri, Sumanlatha Gaddam

**Affiliations:** 1 Bhagwan Mahavir Medical Research Centre, Hyderabad, India; 2 Department of Transplant Biology & Stem Cell, Global Hospital, Hyderabad, India; 3 Department of Genetics, Osmania University, Hyderabad, India; 4 LEPRA India, Blue Peter Public Health & Research Centre, Cherlapally, Hyderabad, India; Public Health Research Institute at RBHS, UNITED STATES

## Abstract

**Background:**

Household contacts of diagnostically established tuberculosis (TB) patients are highly susceptible to disease development. It is surmised that cytokines perhaps play a synergistic and a prognostic role in the activation of the otherwise latent infection in these house hold contacts. Evaluation of the cytokines and any of their inherent polymorphisms might provide a useful diagnostic tool in evaluating the immune regulation and the progression of the disease. The cytokines thus released in a paracrine manner in serum may also provide an indirect measure of the cytokine function.

**Objective:**

The present study was aimed to evaluate the levels of TNF-α, IL-10 & IL-6 cytokines and their correlation with genotype variants amongst tuberculosis patients and their household contacts.

**Methods:**

The cytokine levels were estimated in serum by enzyme-linked immunosorbent assay (ELISA) and their polymorphisms were studied by amplification refractory mutation system polymerase chain reaction (ARMs PCR) in active pulmonary tuberculosis patients (APTB = 150), household contacts (HHC = 190), and healthy controls (HC = 150).

**Results:**

The median values of TNF-α cytokine were significantly high among APTB and HHC compared to HCs (P< 0.0001 and 0.0001). IL-6 levels also were elevated among APTB compared to HHC and HC, and a significant difference was observed between APTB and HHC at P<0.0001; APTB & HC at P< 0.04; HHC & HC at P< 0.01. The IL-10 levels were low in APTB compared to HHC and HCs and no significant difference was observed. TNF-α/IL-10 ratio was significant and indicated Th1 predominance in APTB and HHC. IL-6/IL-10 showed pronounced Th1 expression in APTB and Th2 in HHC and HC. The ROC analysis indicated that both IL-10 and IL-6 can be used to decide the risk of exposed individual to a disease. The results of multivariate analysis indicate that IL-10 (-1082) GA genotype was significantly associated with p<0.028 in APTB. No significant association was observed between genotypes, other serum cytokine levels and clinical characteristics between APTB, HHC and HCs.

**Conclusion:**

Large sample size with follow-up at different time points may further illuminate the role of IL-10 and IL-6 cytokines as a prognostic marker in house hold contacts.

## Introduction

Tuberculosis (TB) caused by *Mycobacterium tuberculosis* (*Mtb*) is a major public health problem worldwide. It is the second leading cause of death from an infectious disease. The world health organization estimated that one third of the world population are infected with *Mtb*, of which only 5–10% advance towards the active disease; while majority of them have latent TB infection (LTBI) [1]. The individuals with latent infection may develop the disease later in their life (reactivation of *Mtb*) or may remain status quo. The most significant source of infection is through aerosol spread of the sputum positive pulmonary tuberculosis patients [2]. The household contacts of index patient are at higher risk of infection ranging from 30% to 80%, depending on the intensity of TB disease transmission [3]. It has become important to identify these high risk group individuals to reduce the disease burden in the community. The pathogenic state of bacterial infection and the probability of reactivation depend on the balance between host immunity and the influence of exogenous factors. During LTBI, *Mtb* exists in a dormant state and later through certain promoting factors such as HIV, malnutrition, tobacco smoke, indoor air pollution, alcoholism, silicosis, insulin dependent diabetes etc., may convert latent form into actively growing bacilli [4]. The host genetic factors have an important role in the progression of the disease. However, a dynamic relationship was proposed between a quiescent and an active state having bidirectional shifts, depending on the load and virulence of the host's immune conditions [5]. The balance between the Th1 and Th2 cytokines reflects the outcome of naïve T cell activation and assists in the elucidation of the immune protection profile of the host against *Mtb*. Certain cytokine gene polymorphisms also correlate with their *in-vitro* cytokine secretion [6]. It is postulated that mutations in the cytokine genes may influence the level of cytokine production and thus the elicited dysregulated immune response [7].

The protective immunity against the pathogen is mediated by cytokines such as IFN-γ, TNF-α, IL-12, IL-6 and IL-18 during the early stage of infection. IFN-γ has been shown to be important for the function and maturation of multiple immune cells [8]. It stimulates macrophages to produce TNF-α which is an essential component of the innate defense mechanism of the host against pathogenic challenge [9]. The production of TNF-α is regulated at the transcriptional and translational level. [10]. The major role of IL-10 is to suppress macrophage and dendritic cell (DC) function, which helps control and initiate the immune responses [11]. During the *Mtb* infection, IL-6 stimulates the secretion of IFN-γ for the activation of macrophages [12]. The production and related cytokine gene polymorphisms may help in the determination of the cytokine secretion and a cascade of pathological events. Therefore, the present study is aimed at understanding the association between cytokine production and their gene polymorphisms in active pulmonary tuberculosis patients and their household contacts for identification of high risk individuals.

## Materials & Methods

### Study group

The present study was carried out during 2009–2012 and the subjects included in the study were from a free chest clinic and PPM-DOTS center, Mahavir Hospital and Research Center. Based on the retrospective data analysis collected during 1998–2008 at our centre, it was observed that most of the TB patients treated were in the age group of 15 to 25 yrs and hence the same age group subjects were included in the study. The household members asymptomatic to TB were taken as contacts. The study included 490 cases of which 150 were active pulmonary tuberculosis patients (APTB), 190 household contacts (HHC), and 150 healthy controls (HC). All patients had positive acid-fast bacilli (AFB) smear microscopy. The bacterial sputum gradation was based on the number of AFB observed on the slide under the microscope as per the Revised National Tuberculosis Control Program (RNTCP) guidelines. Tuberculin skin test (TST) was performed for APTB and HHC and not for HCs.

### Ethical statement

All the study protocols were reviewed and approved by the institutional ethical committee of Bhagwan Mahavir Medical Research Center, Hyderabad, India. Prior written informed consent was taken from all the participants enrolled in the study, and in case of minors, the written consent was taken from their guardians.

### Inclusion criteria

- Patients, household contacts and healthy controls of younger age group of either sex.
- Patients who are sputum positive for pulmonary tuberculosis.
- Patients who are willing to give the consent.

### Exclusion criteria

- Presence of causes of secondary immunodeficiency such as human immunodeficiency virus (HIV), renal transplant, hypertension, diabetes mellitus, malignancy, and cardiac patients.
- Patients unwilling or unable to comply with the study.

## Methodology

### Serum levels of TNF-α, IL-10, IL-6 cytokines

A total of 5 ml blood was collected in red vacutainer and centrifuged at 600 rpm for 10 min. The serum was separated and stored at -80°C in cryovials until analysis. To measure the concentrations of TNF-α, IL-10, IL-6 in serum, an enzyme-linked immunosorbent assay (ELISA) was performed using commercially available kits for cytokine detection (BD Opt EIA for human TNF-α, IL-10, IL-6). The preparation of all reagents, the working standards, and protocol were followed according to the manufacturer's instructions. The absorbance was read using ELISA reader (BIO-RAD) at 450 nm and 570 nm dual filters. The detection ranges for TNF-α, IL-10 were 7.8 pg/ml– 500 pg/ml, & IL-6 was 4.7 pg/ml- 300 pg/ml. All the samples were thawed only once and assayed in duplicate.

### Single nucleotide polymorphism genotyping

**Fig 1:**
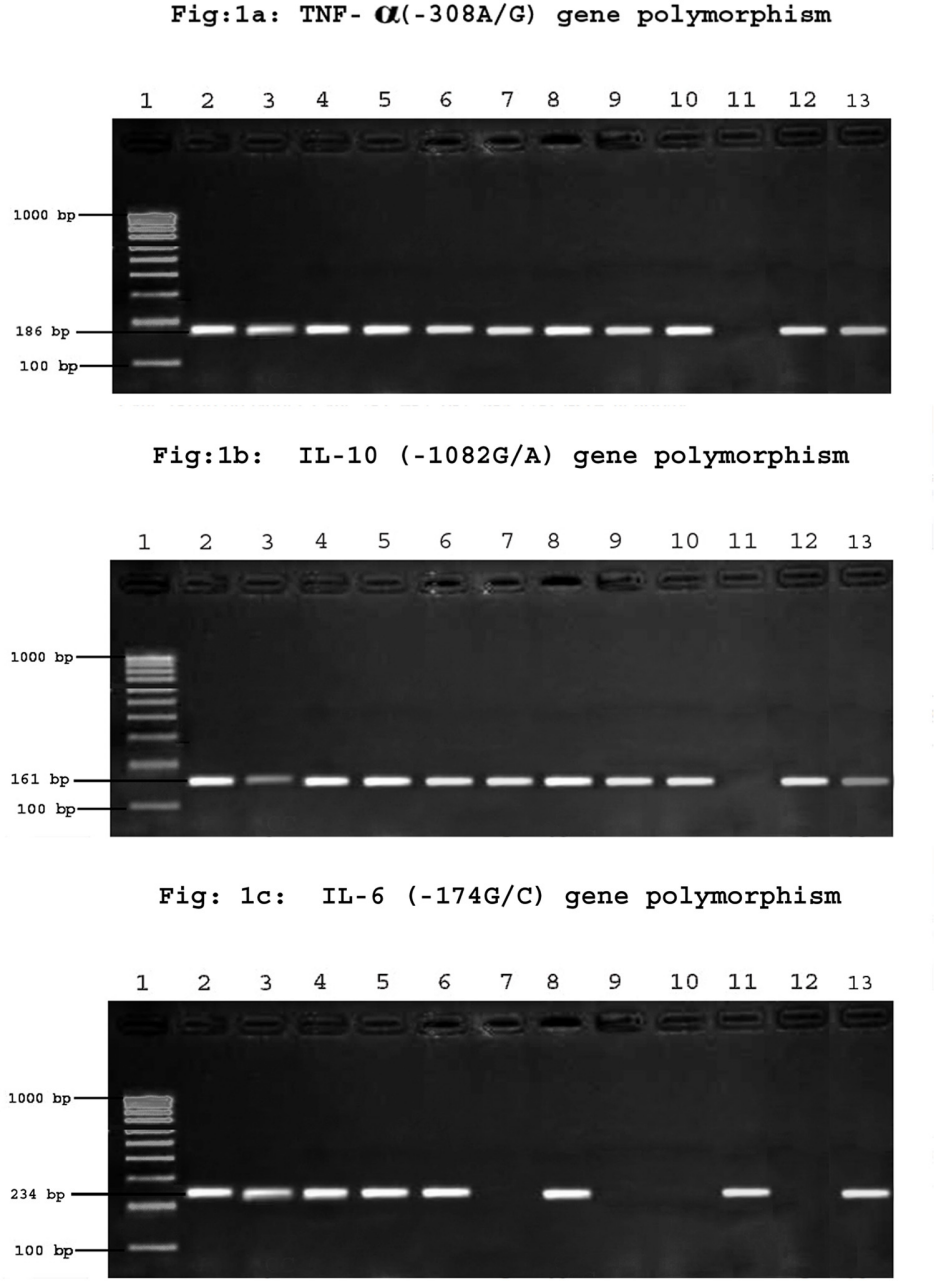
TNF-α (-308A/G), IL-10 (-1082G/A), IL-6 (-174G/C) gene polymorphism. (1a) Lane 1–100 bp ladder;Lane 2–3;4–5;6–7;8-912-13-GA genotype; 10-11-GG genotype; respectively.(1b) Lane 1 – 100bp Ladder; Lane 2 &3 to 8& 9 –GA genotype; Lane 10&11 to 12&13 –GG genotype.(1c) Lane 100- 1000bp ladder;Lane 2-3-GC; 4-5-GC; 6–7,8-9-GG;10-11-CC;12-13-CC genotype.

A 2 ml of whole blood was collected in EDTA tubes for genomic DNA isolation. DNA was extracted from blood using Qiagen kit (Flexi gene DNA isolation kit, Qiagen, Hilden, Germany) according to the manufacturer’s protocol. Quantity of DNA was confirmed by nanodrop and was stored at -20°C for further processing. TNF-α(-308A/G), IL-10(-1082G/A), IL-6(-174G/C) genotyping was carried out by amplification refractory mutation system-polymerase chain reaction (ARMS-PCR) with cycling conditions of 95°C for 8 min followed by 30 cycles at 95°C for 90 s, 60°C for 90 s, 72°C for 1 min and finally for 10 min extension at 72°C. The primers were designed using primer BLAST (NCBI) and were commercially obtained from eurofins (MWG operon). PCR mix (buffer, dNTPs, Taq polymerase) was purchased from Genei Bangalore. TNF- α (308G/A) genotyping was done using common primer 5′-tct cgg ttt ctt ctc cat cg-3′,G allele primer 5′-ata ggt ttt gag ggg cat gg -3′ and A allele primer 5′- ata ggt ttt gag ggg cat ga-3′, IL-10(-1082G/A) genotyping was performed using common primer 5′-tct cgg ttt ctt ctc cat cg-3′,G allele primer 5′-ata ggt ttt gag ggg cat gg -3′ and A allele primer 5′- ata ggt ttt gag ggg cat ga-3′, IL-6(-174G/C) genotyping was carried out using common primer 5′-tct cgg ttt ctt ctc cat cg-3′,G allele primer 5′-ata ggt ttt gag ggg cat gg -3′ and A allele primer 5′- ata ggt ttt gag ggg cat ga-3′. Amplification was carried out using Bio-Rad thermal cycler. Electrophoresis of PCR products was carried out on 2% agarose gel stained with ethidium bromide, and presence or absence of fragments was visualized by UV transilluminator. A product size of 186 bp for TNF- α, 161 bp for IL-10 and 174 bp for IL-6 was deemed a positive amplification (Fig 1a, 1b and 1c).

### Statistical analysis

The cytokine levels were measured by Mann-Whitney test (a non-parametric comparison method for non-normally distributed differences between paired data) using graph pad prism software. Sample median values were used to describe group comparisons since median values are more appropriate for non-normally distributed data. Cytokine ratios were calculated by dividing the total percentage of the respective cytokine. Using IBM SPSS Statistics (version 20), a one-way analysis of variance (ANOVA) with a Tukey posthoc test was used for comparision of 3 groups; multivariate regression analysis was carried out to observe the correlation between gene polymorphisms and serum levels; Receiver-operating-characteristic (ROC) analysis was performed and the areas under the curve (AUC) were obtained for each marker by comparing APTB and HC groups. The optimal cut-off value of each marker was obtained referring to Youden’s *J* statistic (Youden’s idex) as follows: *Youden’s index = sensitivity + specificity– 1*. The maximum of Youden’s index over all maker values is deemed as an optimum cut-off value for the marker. This cut-off was used to determine the high and low risk groups. The analysis was performed using *ROCR* library from *R*– 3.0.0 programming language. The cut-off derived for each marker was used to determine the likelihood of disease susceptibility in a group of individuals in contact along with patient group. A p-value at confidence level 0.05 (two tailed) was considered significant for all the statistical analysis.

## Results

Clinical characteristics were studied in a total of 150 patients, 190 household contacts and 150 healthy controls. For all these subjects age, sex, BCG scar, body mass index (BMI) and tuberculin skin test were taken into consideration for analysis of the data. Males were found to be predominant compared to females in all the three categories and a significant difference was seen in APTB & HHC compared to HC. The mean age of APTB, HHC and HC were (22.74±7.71; 27.67±10.34; 23.49±1.86), and no significant difference was observed between the ages of APTB and HHC when compared to HC. The number of BCG scar negative individuals was more in APTB while in HHC and HC scar positive individuals were high and a significant difference was observed in scar status between the groups. The number of TST-positive individuals was more both in APTB and HHC. The BMI showed a significant difference between the groups (Table 1).

**Table 1:** Clinical characteristic of APTB, HHC and HC.

Clinical characteristics	APTB (n = 150)	HHC (n = 190)	HC (n = 150)	P value APTB vs HC	P value HHC vs HC
Age	22.74±7.71	27.67±10.34	23.49±1.86	Ns	Ns
Sex (M/F)	84/66	130/60	116/34	0.000*	0.000*
BCG(+/-)	38/67	96/37	66/30	0.006*	0.004*
Mean BMI	16.19±2.17	20.91±4.74	24.15±5.49	0.000#	0.000#
TST(+/-)	79/33	97/15	NA	NA	NA

* P value by chi-square analysis;

^#^–significance by T Test

ns- not significant;

NA–not applicable.

### Serum cytokine levels

**Fig 2:**
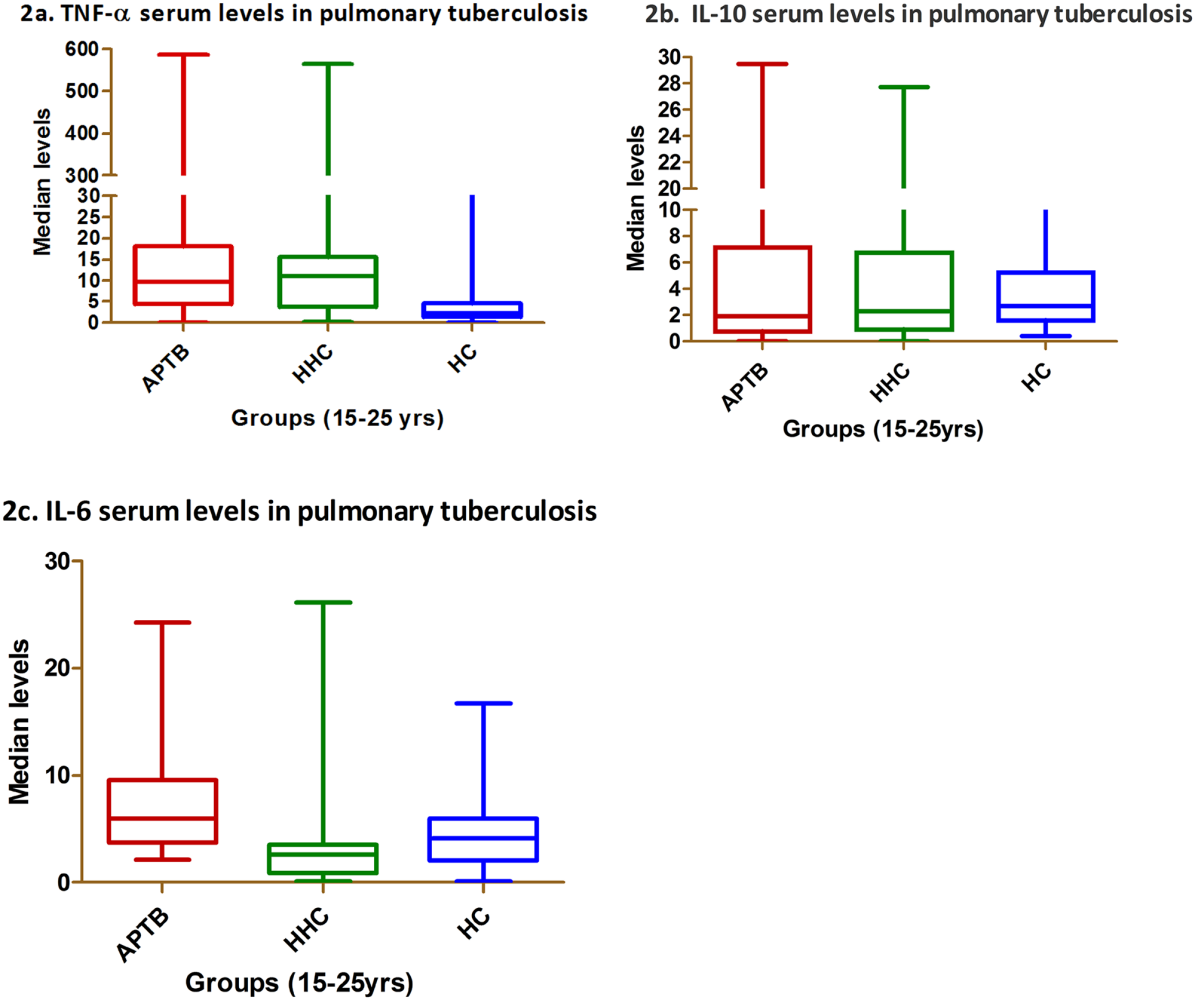
Serum levels of TNF-α, IL-10, IL-6. (2a, 2b, 2c) Data are shown as box plots, where the boxes represent the first through third quartiles, the lines within the boxes represent the median, and the lines outside the boxes represent the minimum and maximum values (excluding outliers). APTB- active pulmonary Tuberculosis patients; HHC-household contacts; HC-healthy controls.

Serum cytokine levels of TNF-α, IL-10 & IL-6 were measured in 130 APTB, 130 HHC &100 HCs [S1 File: elisa levels of TNF- α and IL-10]. The median values of TNF-α cytokine were significantly high among APTB 9.918 pg/ml (IQR 4.51to18.08) and HHC 11.472 pg/ml (IQR 3.70 to 15.59) compared to HCs 2.352 pg/ml (IQR 1.39 to 4.67) (P< 0.0001 and 0.0001) (Fig 2a). The IL-10 levels did not show any significant difference and showed low values in APTB 1.904 pg/ml (IQR 0.77 to 7.10), as compared to HHC 2.303 pg/ml (IQR 0.93 to 6.73) and HCs 2.678 pg/ml (IQR 1.62 to 5.19) (Fig 2b). IL-6 levels also were elevated among APTB 6.044 pg/ml (IQR 3.74 to 9.53) compared to HHC 2.62 pg/ml (IQR 0.86 to 3.48) and HC 4.112 pg/ml (IQR 2.06 to 5.99). A significant difference was observed between APTB and HHC at P<0.0001; APTB &HC at P< 0.04; HHC &HC at P< 0.01 as represented in (Fig 2c).

TNF- α, IL-10 and IL-6 serum levels were high among TST positive household contacts compared to TST negative contacts. The median levels of TST-positive individuals of TNF- α, IL-10 and IL-6 were 11.59 pg/ml, 2.37 pg/ml and 9.50 pg/ml. while the levels of TST negative contacts were 8.43 pg/ml, 1.70 pg/ml, 5.94 pg/ml respectively.

### Ratio of pro-inflammatory and anti-inflammatory cytokines

**Fig 3:**
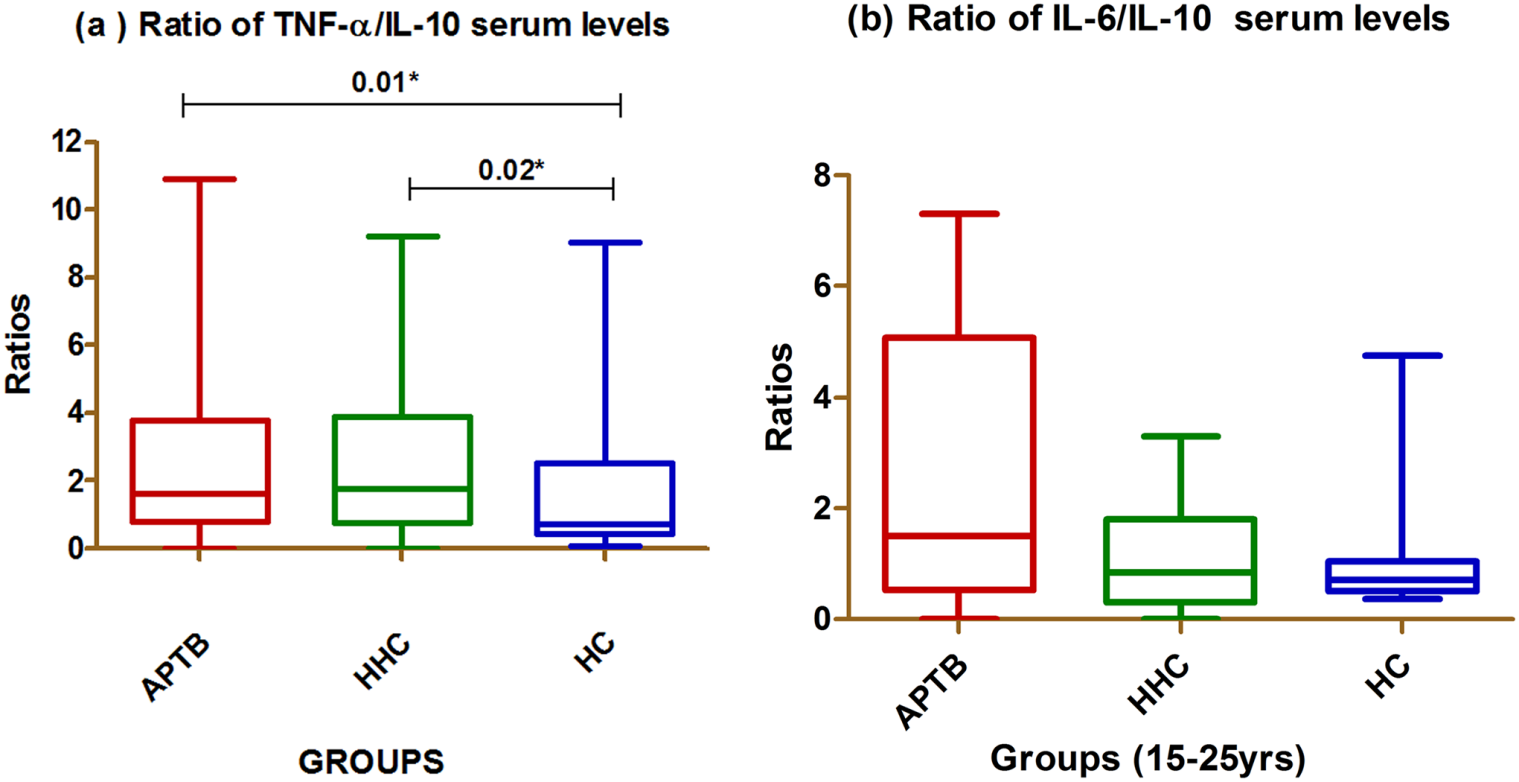
Comparison of cytokines in APTB, HHC and HC categories. (a) Ratio of TNF-α/IL-10 serum levels (b) Ratio of IL-6/IL-10 serum levels. Results are represented as box plot. The threshold for significance was set at p ≤ 0.05. Bars above the plots represent the statistical differences between the groups.

Balance between pro-inflammatory and anti-inflammatory cytokines is important in clinical outcome in several human diseases. TNF- α to IL-10 and IL-6 to IL-10 ratios were calculated in APTB, HHC and HC. Th1 dominance was represented by higher ratio while a lower ratio represented Th2 environment. The median ratio of TNF- α/IL-10 for APTB was 1.628 (IQR 0.79 to 3.78) and for HHC was 1.77 (IQR 0.76to 3.87) suggesting a Th1 environment. In the HCs group, the median value was 0.72 (IQR 0.41 to 2.53) indicating Th2 response. IL-6/IL-10 ratio of APTB was 1.496 (IQR 0.53 to 5.06), HHC was 0.85 (IQR 0.31 to 1.80) and HC 0.71 (IQR 0.50 to 1.05). The IL-6/IL-10 ratio indicates pro-inflammatory response in APTB and an anti-inflammatory response for both HHC and HC. The TNF- α/ IL-10 ratio showed a significant difference between APTB & HC (p<0.01) and HHC & HC (p<0.02). In contrast, IL-6/IL-10 ratio has not show any significance as shown in (Fig 3a and 3b).

The difference between the groups was analyzed using analysis of variance (ANOVA). The result of ANOVA revealed a statistical significant difference at p<0.05 in the TNF-α, IL-10 & IL-6 levels between the subjects. Post hoc comparisons using the Tukey HSD and Bonferroni test also indicated that there was a significant difference in their mean levels between APTB, HHC, and HC as demonstrated in (Table 2).

**Table 2:** ANOVA of TNF-α, IL-10 and IL-6.

Cytokine	Comparisons	Degrees of freedom	F statistic	P value
TNF-α	Between groups	2	4.950	**0.008** *
	Within groups	344		
	Total	346		
IL-10	Between groups	2	8.302	**0.000** *
	Within groups	318		
	Total	320		
IL-6	Between groups	2	4.617	**0.013** *
	Within groups	77		
	Total	79		

*—significant.

### Comparison of cytokine gene polymorphisms of TNF-α (-308A/G), IL-10(-1082G/A) and IL-6(-174G/C) with serum levels

**Fig 4:**
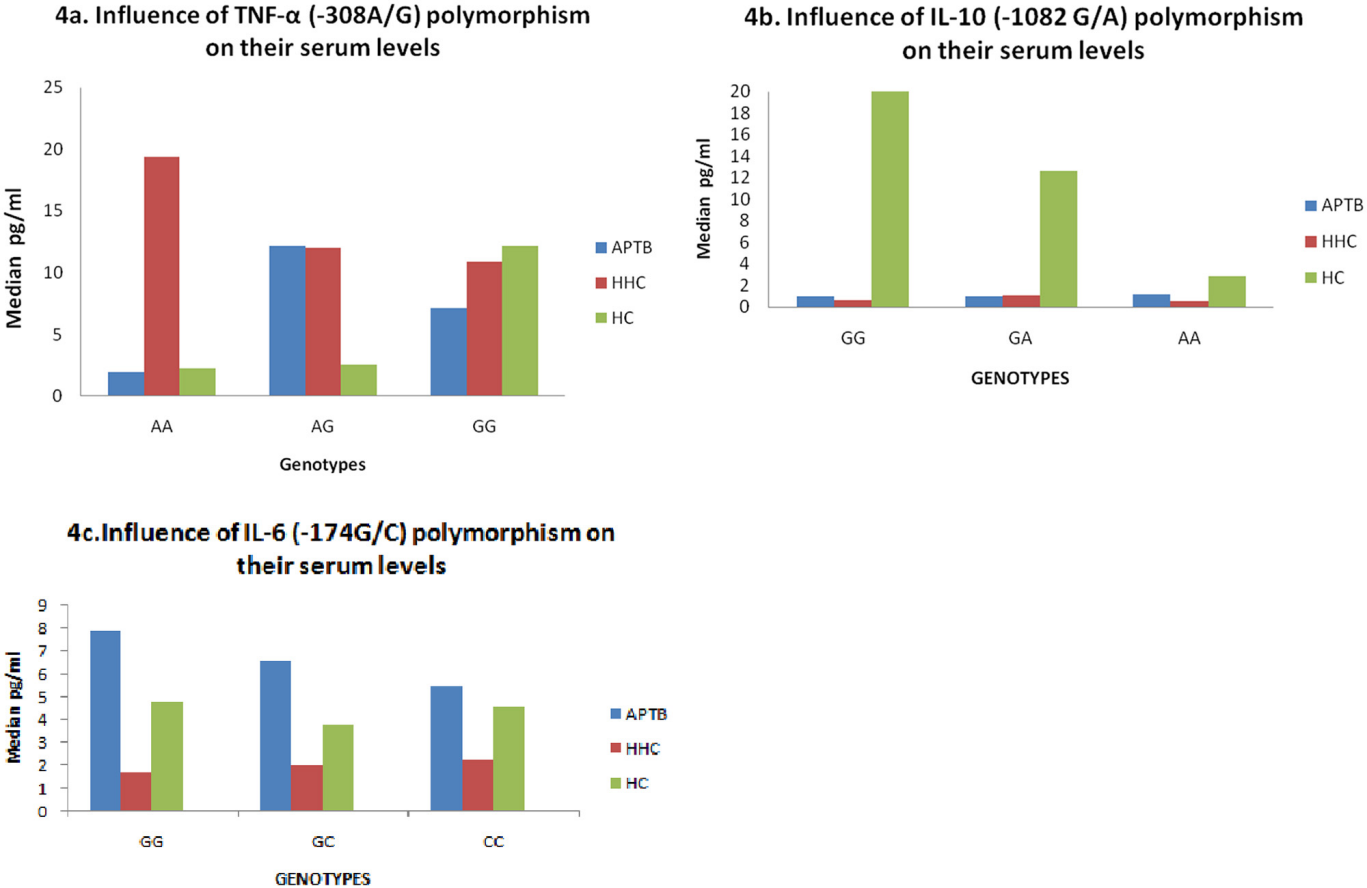
Comparision of cytokine gene polymorphisms of TNF-α (-308A/G), IL-10(-1082G/A) and IL-6(-174G/C) with serum levels. (4a, 4b, 4c) Vertical axis- serum levels showing median values; Horizontal axis- genotypes of TNF- α (-308A/G); IL-10(-1082G/A) and IL-6(-174G/C),APTB—active pulmonary tuberculosis patients; HHC-household contacts; HC-healthy controls.

The AG genotype showed high levels of TNF-α (-308A/G) in serum among APTB and HHC when compared with HC at P<0.0002 &P<0.0006. The GA genotype of IL-10 were showing significantly low serum levels in APTB & HHC compared to HC at P<0.002 & P<0.001. The AA genotype was also a low producer in HHC compared to APTB &HC and was significant at P<0.005. The CC genotype of IL-6 was a high producer than other genotypes in APTB compared to HHC and HC & was significant at P<0.02 (Fig 4a, 4b and 4c).

### Multivariate analysis correlating serum levels their clinical characteristics and single nucleotide polymorphisms

To evaluate the possible association of TNF-α, IL-10 & IL-6 between cytokine gene polymorphisms and the serum levels, multivariate analysis was performed with BMI as a covariate in APTB, HHC & HC. It was found that IL-10 GA genotype was significantly associated in APTB in comparison to HCs with p<0.028, whereas no significant association was found between the genotype and the serum levels of TNF-α & IL-6 cytokines (p>0.05). There was no significant association between clinical characteristics and their serum levels between APTB, HHC and HCs.

### ROC analysis

The ROC curves were obtained by comparing APTB and HC for the three markers TNF-α, IL-10 and IL-6 (Table 3). The area under the curve along with 95% confidence interval was obtained along with statistical significance. Further, the cut-off value corresponding to maximum Youden’s index was derived to determine the high and low risk groups with reference to each marker. Table 3 reveals that for TNF-α, the cut-off obtained was 20.98 corresponding to maximum index of 1.27. The area under the curve (AUC) was 0.51 with a *p*-value of 0.851 (*p*> 0.05), which prompts us to accept the null hypothesis that AUC is 0.5. In other words, the marker is less likely to contribute to the separation of study groups.

**Table 3:** Comparison of TNF- α, IL-10 and IL-6 between patient (APTB) and Control (HC) groups.

Parameter	AUC	95% CI: AUC	P-value	Max (J statistic)	Cut-off	Accuracy
TNF- α	0.510	0.420–0.597	0.851	1.27	20.98	0.526
IL-10	0.668	0.575–0.761	**< 0.0001** *	1.31	**1.71**	0.550
IL- 6	0.708	0.561–0.856	**0.006** *	1.54	**7.83**	0.769

*- significant p<0.05

AUC- area under the curve.

For IL-10, the maximum Youden’s statistic obtained was 1.31 and the corresponding cut-off value was 1.71. The area under the curve (AUC) was 0.668, indicated by *p*-value < 0.001. The maximum Youden’s statistic for IL-6 was 1.54 and the corresponding cut-off value was 7.83. The area under the curve (AUC) was 0.708 with a *p*-value of 0.006.

**Fig 5:**
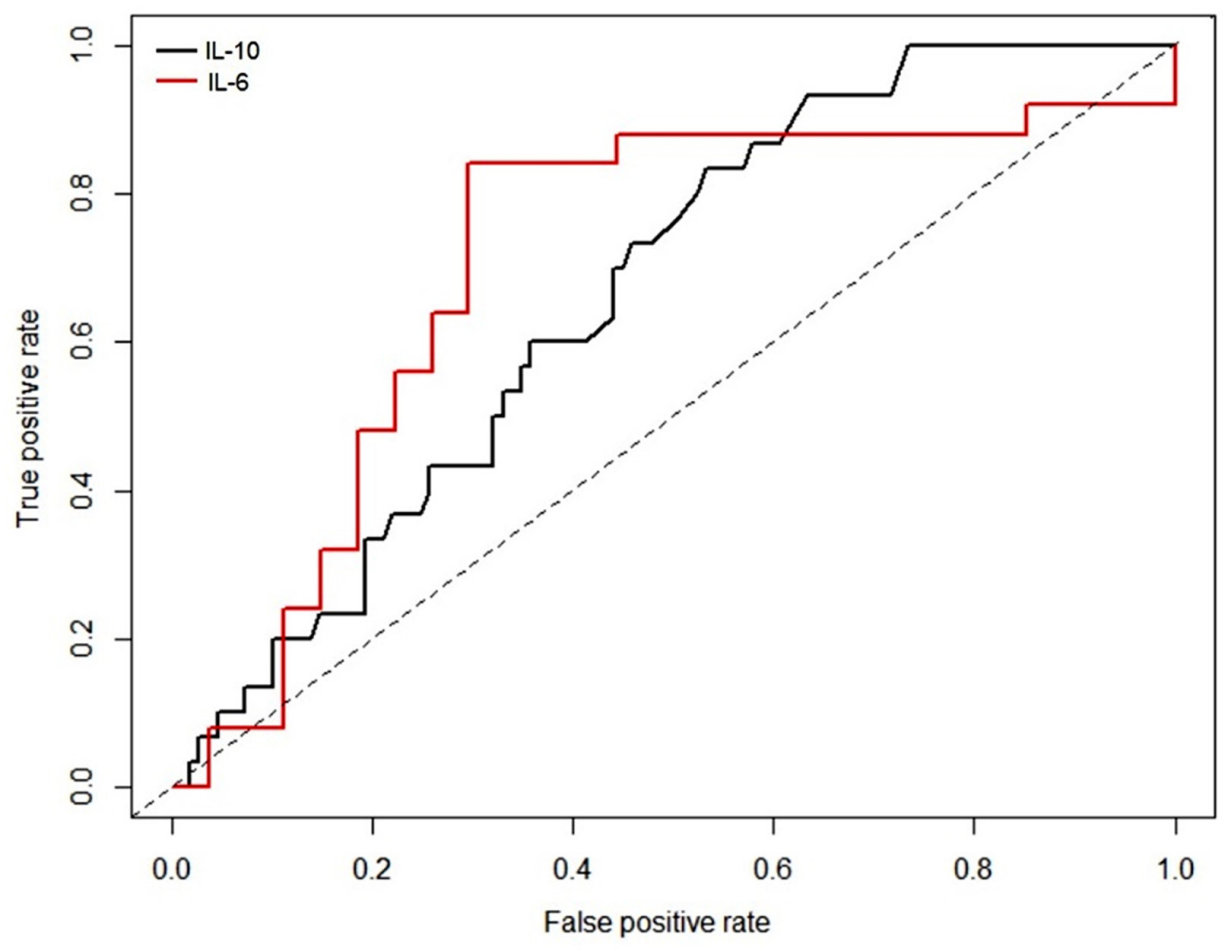
ROC plots for IL-10 and IL-6 for the comparison between patient (APTB) and control (HC) groups.

Thus, it is evident that amongst the three parameters, the AUC corresponding to IL-10 and IL-6 were significantly different than 0.5, indicating that these markers could provide discrimination between APTB and HC. Hence, the ROC plots were obtained for these two markers as shown in (Fig 5).

As regards IL-10, the subjects with IL-10 less than 1.71 were considered as high risk, while those above this threshold was at low risk. Similarly, for IL-6, the subjects with IL-6 less than 7.83 were considered at high risk, while those above the threshold was considered at low risk.

The HHCs were evaluated for risk of disease based on IL-10 and IL-6 values and referring to the respective cut-offs of the markers.

An event E was defined as ‘marker value less than the cut-off’ derived from the ROC analysis. If D indicates the disease group and C the contact group, then the probability that an individual in contact with patient could be a disease case is given by:

P(individual in contact with patient has a disease) =P(E / C)×P(D / E)

Alternately, the probability is the product of probability that the contact person has marker values less than the threshold. The probability of a given disease is the marker value that which is less than the threshold.

For each marker, these probabilities were evaluated and the likelihood of disease for a person in contact with patient was determined. Based on the IL-10 values from APTB and HC, for the given cut-off 1.71, the probability that a person with IL-10 less than 1.71 has a disease is 0.91 (101/110), and the probability of being normal is 0.081 (9/110). In other words, *P* (D/E) is 0.91 and *P* (N/E) is 0.081.

In the HHC group, the probability of finding an individual with IL-10 less than 1.71 was 0.444 (49/110). That is, *P* (E/C) was 0.444. Therefore, the probability that an individual in contact with patient could have disease is 0.404 as per above expression. Nearly 40% of the exposed individuals with IL-10 value less than 1.71 could have disease. The probability that the person from contact group could be normal is 0.035 (0.444×0.081) i.e. 4% of individuals from contact group with IL-10 less than 1.71 could be normal.

Similarly, based on the IL-6 data from APTB and HC, and for the given cut-off of 7.83, the probability that a person with IL-6 less than 7.83 has a disease is 0.8272 (91/110), and the probability of being normal is 0.1727 (19/110). In other words, *P* (D/E) is 0.8272 and *P* (N/E) is 0.1727.

In the HHC group, the probability of finding an individual with IL-6 less than 7.98 was 0.9545 (105/110). That is, *P* (E/C) was 0.9545. Therefore, the probability that an individual in contact with patient could have disease is 0.7895. In other words, nearly 79% of the individuals in contact with patients and IL-6 value less than 7.83 could have disease. The probability that the person from contact group could be normal is 0.165 (0.9545×0.1727), i.e. 16% of the individuals from contact group with IL-6 less than 7.83 could be normal.

The positive prediction value (PPV) of the cut-off for IL-10 (0.91) was higher than that of IL-6 (0.8272), indicating a better diagnostic strength of IL-10 in detecting high risk candidates as compared to IL-6. On the contrary, the negative prediction value (NPV) of the cut-off for IL-10 (0.30) was smaller than that of IL-6 (0.72), indicating better diagnostic strength of the later in detecting low risk candidates as compared to Il-10. So both of these tests could be used in conjunction to decide the risk of exposed individual to a disease. Moreover, both the markers have significant AUCs, with IL-6 having slightly higher than IL-10.

Correlation analysis did not show any significant relationship between the studied serum cytokine levels and the clinical characteristics in APTB, HHC & HC.

## Discussion

TNF-α contributes to the pathogenesis of tuberculosis due to its role in the formation and maintenance of granulomas [13]. TNF-α is considered necessary to remove bacteria in inflammatory lesions and hence TNF- α has been found to be one of the important cytokines in the control of *Mtb*. infection [14][15].

In our study, elevated levels of serum TNF- α have been reported in APTB and HHC compared to HCs. To best of our knowledge, this is the first study to report serum levels in household contacts of TB patients in south India. A Chinese study showed elevated levels of TNF- α in patients than in controls [16]. Several other studies reported increased levels of TNF- α in serum of TB patients [17][18][19][20]. An Ethiopian study showed undetectable levels of TNF- α in plasma of TB patients and have stated that it may be due to the suppressive effect of TGF-β [21]. In contrast to our results a study from North India reported decreased serum levels of TNF- α in patients [22]. Our results are in agreement with most of the reports that TNF- α serum levels were high in TB patients than in controls. TNF- α contributes both to the protection against tuberculosis and to immunopathology. Despite the high levels of TNF-α in patient, the disease occurrence may be due to marked tissue necrosis leading to progressive TB and may result in the release of TNF- α into the circulation contributing to systemic indicators of TB, such as fever and cachexia. In household contacts, the high TNF-α level indicate their release by NK cells in early stages i.e. between 0–4 weeks and the cytokine balance may aid in disease suppression.

IL-10 which is a T regulatory cytokine plays a central role during chronic and latent stage of pulmonary TB [23]. The IL-10 production is high during the infection promoting reactivation of TB. The excessive production of this cytokine results in failure to control the infection. Many studies have reported the increased production of IL-10 in patients with active disease. There are few reports of contacts with IL-10 production reporting elevated levels of IL-10 in plasma of the contacts compared to patients [21]. A Taiwan study described the high IL-10 production in patients compared to controls [24]. Similarly in our study, we observed high production of IL-10 in APTB and HHC compared to HC. Another recent study [25] reported that the interferon gamma release assay (IGRA) negative contacts showed high IL-10 serum levels than IGRA positive contacts and patients at diagnosis. In other diseases like depression and multiple sclerosis, low IL-10 production was observed which was reported by several other groups [26][27]. Though the IL-10 levels are high in contacts, they do not progress to infection. The reason may be that the high pro-inflammatory response of TNF-α may be inhibited by high IL-10 production, which might be involved in natural defense resulting in Th1-Th-2 balance being beneficial to these contacts. Whereas, high IL-10 production in patient, suppresses immune response leading to inadequate balance of pro and anti- inflammatory cytokines.

*Mtb* and its components have been shown to stimulate mononuclear phagocytes *in vitro* to release IL-6. When it reaches the systemic circulation it causes symptoms such as fever, malaise, and weight loss. The synthesis of this cytokine may be induced by infectious agents and other cytokines such as IL-1 and TNF-α [28].

Studies have reported that mononuclear cells from early-active TB patients can up-regulate pro-inflammatory cytokines, such as IL-6. In our study, we have found high serum levels of IL-6 in APTB compared to HHC and HC. Similar results were obtained in serum from pulmonary tuberculosis patients compared to healthy controls [29][30]. Another study also reported increased IL-6 in subjects with active TB disease compared to those with latent tuberculosis infection [31]. An Asian study showed high IL-6 concentrations in patients with pulmonary cavities than in patients without cavities signifying the disease severity [32]. Another Chinese study also reported increased IL-6 concentration in active TB compared with Latent TB infections, which were family contacts [25]. The increased IL-6 concentration in patients and household contacts when compared with controls may be due to release of IL-6 into circulation during early stages of infection causing systemic symptoms and hence the levels may also vary depending upon the clinical status of the patients or contacts. As stated, earlier production of IL-6 is to a large extent under the control of TNF-α and IL-1, and thus its concentration in blood may reflect local production of these cytokine in the lungs.

## Conclusion

The results of the present study indicate the association of these cytokines with the disease. Based on the genotype association and their production IL-10 gene were found to be significantly associated with the disease. IL-6/IL-10 ratio may be used to discriminate the HHCs from APTB. The AG genotype of TNF-α and GA of IL-10 were significantly associated with the disease. The association between the SNPs and their cytokine production indicate the functional importance of cytokines in gene expression. The ROC results suggest that IL-10 and IL-6 may be used as a biomarker to differentiate between APTB, HHC, and HCs. In conclusion the serum cytokine levels of IL-10 and IL-6 may be used as a suitable biomarker for the identification of high risk individuals.

Further, follow-up studies in large sample size might help in identifying a specific cytokine as a biomarker to determine the high risk individuals towards the progression of the disease.

## Supporting Information

S1 File ELISA levels of TNF-α and IL-10. The file contains ELISA levels of TNF-α and IL-10 in APTB, HHC and HC. Column (A) & (C) indicates names of patients and their contacts; B, D &E contains the ELISA levels.
(PDF)
