# Correction: Evaluation of TNF-α, IL-10 and IL-6 Cytokine Production and Their Correlation with Genotype Variants amongst Tuberculosis Patients and Their Household Contacts

**DOI:** 10.1371/journal.pone.0317538

**Published:** 2025-01-09

**Authors:** The PLOS ONE Editors

## Notice of Republication

This article [1] was republished on November 19, 2024, to address an issue identified post-publication. Please download this article again to view the correct version.

The article’s Data Availability statement is updated to: All relevant data are within the paper and its Supporting Information files.
