# Correction: Evaluation of TNF-α, IL-10 and IL-6 Cytokine Production and Their Correlation with Genotype Variants amongst Tuberculosis Patients and Their Household Contacts

**DOI:** 10.1371/journal.pone.0318949

**Published:** 2025-02-04

**Authors:** Lavanya Joshi, Meenakshi Ponnana, Ramya Sivangala, Lakshmi Kiran Chelluri, Prathiba Nallari, Sitaramaraju Penmetsa, Vijayalakshmi Valluri, Sumanlatha Gaddam

There is an error in the S1 File legend. The correct legend is:

## Supporting information

S1 File ELISA levels of TNF-α and IL-10. The file contains ELISA levels of TNF-α and IL-10 in APTB, HHC and HC. Column (A), (C) & (E) indicates APTB, HHC and HC; B, D & F contains the ELISA levels.
(PDF)
