# Supplementary material for: Evaluation of TNF-α, IL-10 and IL-6 Cytokine Production and Their Correlation with Genotype Variants amongst Tuberculosis Patients and Their Household Contacts
Source: PLoS One. 2015 Sep 11;10(9):e0137727. doi: 10.1371/journal.pone.0137727 (PMC4567353; doi:10.1371/journal.pone.0137727)
Supplement: S1 File — The file contains ELISA levels of TNF-α and IL-10 in APTB, HHC and HC. Column (A) & (C) indicates names of patients and their contacts; B, D &E contains the ELISA levels. (PDF) [file pone.0137727.s001.pdf]

TNF-α LEVELS					
APTB		HHC		HC	
1	35.958	1	15.726	1	1.855
2	7.89	2	3.704	2	2.352
3	282.88	3	3.789	3	1.461
4	4.955	4	6.117	4	1.461
5	4.862	5	5.049	5	1.336
6	13.352	6	12.715	6	1.098
7	12.969	7	13.999	7	1.156
8	0.041	8	11.964	8	1.336
9	12.088	9	11.964	9	1.275
10	25.263	10	23.549	10	3.451
11	23.858	11	12.463	11	1.398
12	7.142	12	3.535	12	1.275
13	2.134	13	1.398	13	2.134
14	10.27	14	9.801	14	1.654
15	10.035	15	35.08	15	1.855
16	2.426	16	1.72	16	3.535
17	84.917	17	43.791	17	2.426
18	12.463	18	11.594	18	4.677
19	1.787	19	1.461	19	1.855
20	1.336	20	0.143	20	4.137
21	28.311	21	34.905	21	9.801
22	1.787	22	2.063	22	3.535
23	1.654	23	0.316	23	1.398
24	16.959	24	21.725	24	2.576
25	6.829	25	1.275	25	4.677
26	1.524	26	1.461	26	1.156
27	2.426	27	2.729	27	1.098
28	32.658	28	12.338	28	1.654
29	1.924	29	1.993	29	2.807
30	1.72	30	0.241	30	4.05
31	3.619	31	2.134	31	7.998
32	1.72	32	1.461	32	2.063
33	1.524	33	1.993	33	2.807
34	35.08	34	14.392	34	1.993
35	0.207	35	1.654	35	11.594
36	0.174	36	1.72	36	9.918
37	33.172	37	28.311	37	16.545
38	11.594	38	18.506	38	4.77
39	18.935	39	12.842	39	13.999
40	20.388	40	16.959	40	16.407
41	14.788	41	17.657	41	18.079
42	0.143	42	2.279	42	22.026
43	2.652	43	1.524	43	9.34
44	1.04	44	1.72	44	13.096

IL-10 LEVELS					
APTB		HHC			HC
1	6.105	1	72.208	1	
2	8.905	2	20.324	2	0.562
3	0.031	3	1.709	3	1.904
4	0.25	4	(*)	4	4.36
5	0.069	5	0.299	5	50.476
6	11.244	6	15.42	6	38.14
7	(*)	7	0.155	7	21.906
8	0.155	8	1.969	8	15.687
9	6.339	9	6.339	9	11.755
10	11.159	10	8.009	10	3.992
11	0	11	0.031	11	17.303
12	4.139	12	145.021	12	(*)
13	0.674	13	1.969	13	33.814
14	15.598	14	9.648	14	1.58
15	0.788	15	2.235	15	2.169
16	6.028	16	10.484	16	2.575
17	(*)	17	(*)	17	3.131
18	0.402	18	0.402	18	2.102
19	7.767	19	0.508	19	2.102
20	1.266	20	0.562	20	2.036
21	1.144	21	1.144	21	2.37
22	0.963	22	0.402	22	9.814
23	9.814	23	41.407	23	3.919
24	0.202	24	4.508	24	4.065
25	0.031	25	3.558	25	33.714
26	0.904	26	1.083	26	4.582
27	3.201	27	2.303	27	31.335
28	0.25	28	1.904	28	3.629
29	0.454	29	0.35	29	11.499
30	1.205	30	2.712	30	11.841
31	45.44	31	7.286	31	10.4
32	1.328	32	1.773	32	5.489
33	0.846	33	2.303	33	6.968
34	1.969	34	4.434	34	4.807
35	6.183	35	1.644	35	7.526
36	1.391	36	4.286	36	2.781
37	17.938	37	(*)	37	35.415
38	1.266	38	0.963	38	38.039
39	0.202	39	0.069	39	14.534
40	16.582	40	13.569	40	33.615
41	1.391	41	2.506	41	130.46
42	0.155	42	0.562	42	74.776
43	1.709	43	2.99	43	47.424
44	(*)	44	1.328	44	58.816

TNF-α LEVELS					
APTB		HHC		HC	
**45**	3.124	**45**	2.063	**45**	118.821
**46**	376.72	**46**	602.563	**46**	32.317
**47**	1.72	**47**	1.924	**47**	0.114
**48**	1.336	**48**	1.654	**48**	15.054
**49**	1.215	**49**	6.017	**49**	33.344
**50**	16.683	**50**	10.986	**50**	7.353
**51**	17.237	**51**	14.13	**51**	10.388
**52**	15.054	**52**	15.591	**52**	34.209
**53**	3.451	**53**	2.206	**53**	15.998
**54**	2.134	**54**	1.855	**54**	2.279
**55**	6.726	**55**	13.096	**55**	1.855
**56**	483.02	**56**	447.416	**56**	2.576
**57**	5.721	**57**	4.955	**57**	2.964
**58**	5.43	**58**	19.657	**58**	1.787
**59**	11.717	**59**	16.959	**59**	1.083
**60**	5.526	**60**	16.821	**60**	0.34
**61**	548.33	**61**	1095.38	**61**	0
**62**	675.78	**62**	529.046	**62**	4.562
**63**	1.215	**63**	1.215	**63**	2.863
**64**	2.206	**64**	2.501	**64**	1.879
**65**	3.619	**65**	4.586	**65**	0.479
**66**	32.317	**66**	13.096	**66**	2.375
**67**	12.969	**67**	12.969	**67**	0.987
**68**	1.461	**68**	1.098	**68**	2.548
**69**	30.963	**69**	15.322	**69**	0.581
**70**	586.27	**70**	471.323	**70**	1.535
**71**	552.56	**71**	416.166	**71**	0.804
**72**	11.472	**72**	13.352	**72**	1.959
**73**	13.869	**73**	17.098	**73**	0.529
**74**	481.4	**74**	554.248	**74**	0.833
**75**	507.92	**75**	564.859	**75**	2.955
**76**	536.15	**76**	782.448	**76**	1.647
**77**	552.13	**77**	562.731	**77**	2.863
**78**	659.16	**78**	431.31	**78**	1.61
**79**	543.7	**79**	541.18	**79**	1.535
**80**	11.84	**80**	9.111	**80**	1.116
**81**	13.869	**81**	15.188	**81**	3.431
**82**	6.318	**82**	4.586	**82**	2.505
**83**	9.111	**83**	17.516	**83**	1.462
**84**	8.773	**84**	20.683	**84**	0.924
**85**	16.134	**85**	14.788	**85**	1.801
**86**	15.456	**86**	12.088	**86**	0.504
**87**	4.05	**87**	6.623	**87**	5.112
**88**	4.955	**88**	6.521	**88**	3.529

IL-10 LEVELS					
APTB		HHC			HC
**45**	0.111	**45**	0.202	**45**	82.33
**46**	2.37	**46**	0.508	**46**	76.346
**47**	0.788	**47**	0.788	**47**	63.49
**48**	0.674	**48**	0.562	**48**	49.104
**49**	0.674	**49**	4.36	**49**	64.037
**50**	5.95	**50**	1.083	**50**	36.925
**51**	0.031	**51**	3.846	**51**	42.024
**52**	2.575	**52**	24.359	**52**	33.914
**53**	54.944	**53**	48.894	**53**	59.681
**54**	10.316	**54**	9.648	**54**	47.738
**55**	1.266	**55**	9.648	**55**	84.377
**56**	10.99	**56**	13.395	**56**	23.79
**57**	0.111	**57**	(*)	**57**	90.331
**58**	1.266	**58**	0.788	**58**	73.77
**59**	0.562	**59**	0.202	**59**	70.429
**60**	15.687	**60**	27.621	**60**	58.708
**61**	2.99	**61**	1.517	**61**	3.846
**62**	9.399	**62**	15.065	**62**	0.588
**63**	0.111	**63**	0.299	**63**	0.387
**64**	2.781	**64**	2.851	**64**	5.318
**65**	1.904	**65**	2.506	**65**	23.541
**66**	2.851	**66**	26.367	**66**	0.419
**67**	0.618	**67**	0.299	**67**	7.734
**68**	2.643	**68**	3.486	**68**	2.563
**69**	5.032	**69**	5.873	**69**	1.711
**70**	0.069	**70**	0.618	**70**	1.468
**71**	12.701	**71**	9.898	**71**	0.624
**72**	8.171	**72**	11.841	**72**	3.959
**73**	0.069	**73**	0.155	**73**	5.389
**74**	1.969	**74**	8.09	**74**	94.063
**75**	0	**75**	2.303	**75**	1.661
**76**	(*)	**76**	0	**76**	1.811
**77**	(*)	**77**	0.202	**77**	0.588
**78**	1.58	**78**	1.205	**78**	5.46
**79**	0.25	**79**	0.35	**79**	40.779
**80**	1.328	**80**	0.788	**80**	3.83
**81**	(*)	**81**	0.069	**81**	1.612
**82**	0.73	**82**	3.272	**82**	5.604
**83**	0.069	**83**	1.58	**83**	1.237
**84**	(*)	**84**	2.036	**84**	1.515
**85**	0.73	**85**	1.083	**85**	2.231
**86**	1.083	**86**	0.069	**86**	1.192
**87**	1.58	**87**	2.303	**87**	60.403
**88**	(*)	**88**	1.904	**88**	38.517

TNF-α LEVELS					
APTB		HHC		HC	
**89**	25.105	**89**	7.353	**89**	
**90**	25.578	**90**	12.088	**90**	
**91**	13.61	**91**	13.739	**91**	
**92**	19.803	**92**	15.591	**92**	
**93**	22.784	**93**	11.35	**93**	
**94**	24.792	**94**	19.367	**94**	
**95**	4.05	**95**	4.05	**95**	
**96**	4.405	**96**	4.226	**96**	
**97**	21.875	**97**	12.088	**97**	
**98**	22.936	**98**	10.865	**98**	
**99**	18.363	**99**	13.096	**99**	
**100**	26.054	**100**	18.363	**100**	
**101**	4.955	**101**	6.017	**101**	
**102**	7.037	**102**	14.921	**102**	
**103**	6.017	**103**	3.704	**103**	
**104**	14.921	**104**	12.715	**104**	
**105**	15.054	**105**	15.456	**105**	
**106**	7.142	**106**	16.545	**106**	
**107**	6.726	**107**	8.605	**107**	
**108**	6.521	**108**	9.454	**108**	
**109**	7.247	**109**	9.685	**109**	
**110**	5.623	**110**	9.111	**110**	
**111**	9.454	**111**	8.108	**111**	
**112**	8.217	**112**	8.998	**112**	
**113**	6.623	**113**	8.438	**113**	
**114**	9.801	**114**	3.535	**114**	
**115**	6.017	**115**	8.549	**115**	
**116**	8.108	**116**	20.979	**116**	
**117**	12.842	**117**	10.035	**117**	
**118**	11.594	**118**	7.46	**118**	
**119**	9.569	**119**	8.108	**119**	
**120**	8.998	**120**	13.224	**120**	
**121**	14.656	**121**	10.507	**121**	
**122**	12.338	**122**	1.461	**122**	
**123**	11.107	**123**	12.589	**123**	
**124**	9.569	**124**	32.146	**124**	
**125**	7.998	**125**	12.969	**125**	
**126**	10.865	**126**	5.721	**126**	
**127**	10.27	**127**	8.108	**127**	
**128**	10.152	**128**	14.261	**128**	
**129**	6.726	**129**	6.117	**129**	
**130**	5.143	**130**	13.481	**130**	

IL-10 LEVELS					
APTB		HHC			HC
**89**	3.774	**89**	9.731	**89**	2.395
**90**	2.643	**90**	1.328	**90**	10.679
**91**	6.889	**91**	9.482	**91**	1.148
**92**	1.266	**92**	1.773	**92**	1.148
**93**	6.339	**93**	3.131	**93**	1.061
**94**	20.324	**94**	0	**94**	0.853
**95**	1.328	**95**	1.453	**95**	0.853
**96**	2.575	**96**	4.882	**96**	2.177
**97**	8.252	**97**	16.223	**97**	(*)
**98**	15.42	**98**	10.99	**98**	1.192
**99**	28.009	**99**	33.814	**99**	4.828
**100**	8.496	**100**	15.153	**100**	1.018
**101**	1.58	**101**	1.328	**101**	1.192
**102**	29.469	**102**	52.49	**102**	36.235
**103**	1.023	**103**	0.963	**103**	11.315
**104**	2.235	**104**	2.575	**104**	5.13
**105**	1.453	**105**	2.438	**105**	8.306
**106**	10.652	**106**	7.767	**106**	1.595
**107**	10.736	**107**	9.482	**107**	0.838
**108**	2.102	**108**	3.629	**108**	1.023
**109**	1.773	**109**	2.303	**109**	1.155
**110**	1.391	**110**	8.905	**110**	1.343
**111**	9.317	**111**	13.047	**111**	1.001
**112**	11.755	**112**	27.718	**112**	1.001
**113**	10.064	**113**	5.642	**113**	0.98
**114**	2.303	**114**	2.438	**114**	1.088
**115**	85.517	**115**	358.047	**115**	4.147
**116**	2.99	**116**	5.184	**116**	1.622
**117**	1.904	**117**	0.904	**117**	1.675
**118**	2.575	**118**	1.969	**118**	20.4
**119**	1.969	**119**	7.127	**119**	1.868
				**120**	18.421
				**121**	1.517
				**122**	4.997
				**123**	5.175
				**124**	4.436
				**125**	2.221
